# Timing of 30-Day Hospital Readmission Risk and Its Implications for Clinical Utility

**DOI:** 10.1001/jamanetworkopen.2026.28846

**Published:** 2026-08-13

**Authors:** Mohamad Zafer Alkayali, Yuelei Fu, Janna C. Castro, Matt Gill, Shant Ayanian, Sagar B. Dugani

**Affiliations:** 1 Division of Hospital Internal Medicine, Mayo Clinic, Rochester, Minnesota; 2 Center for Digital Health, Mayo Clinic, Rochester, Minnesota; 3 Department of Information Technology, Mayo Clinic, Rochester, Minnesota; 4 Robert D. and Patricia E. Kern Center for the Science of Health Care Delivery, Mayo Clinic, Rochester, Minnesota

## Abstract

**Importance:**

Thirty-day hospital readmission is used to guide postdischarge care; however, a single binary 30-day estimate does not distinguish when risk occurs from when intervention is most actionable, with implications for allocation of postdischarge resources.

**Objective:**

To evaluate time-structured hospital readmission risk within 30 days after discharge and implications for clinical utility.

**Design, Setting, and Participants:**

Retrospective prognostic study of adults with hospital discharges from 2017 to 2024 (from 19 hospitals within a US health system for the development cohort) and 2008 to 2019 (from Medical Information Mart for Intensive Care IV for the external cohort) with 30-day follow-up. Analyses were conducted from November 2025 through June 2026.

**Exposure:**

Postdischarge prediction window, including landmark intervals of 0 to 7, 8 to 14, 15 to 21, and 22 to 30 days and cumulative horizons through days 7, 14, 21, and 30 after discharge.

**Main Outcomes and Measures:**

First all-cause hospital readmission before death during each landmark interval and by each cumulative horizon. Risk estimates were derived using clinical information available at hospital discharge. Performance was assessed using discrimination, calibration, decision curves, and capacity-constrained analyses.

**Results:**

Among 617 841 discharges in the development cohort, the median (IQR) patient age was 65 (50-76) years, and 315 220 discharges (51.0%) occurred among female patients. The 30-day readmission rate was 16.3% (95% CI, 16.2%-16.5%), with 68 797 readmissions (68.2%) occurring within 14 days. Cumulative-horizon analyses characterized readmission risk through days 7, 14, 21, and 30, whereas landmark interval-specific analyses showed lower positive predictive values (PPVs) beyond day 7. At 5% intervention capacity, landmark PPVs were 58.3% (95% CI, 57.4%-59.1%) for days 0 to 7, 16.0% (95% CI, 15.5%-16.6%) for days 8 to 14, 14.5% (95% CI, 13.9%-15.0%) for days 15 to 21, and 12.9% (95% CI, 12.4% to 13.5%) for days 22 to 30; numbers needed to prevent 1 readmission were 8.6, 31.3, 34.6, and 38.6, respectively.

**Conclusions and Relevance:**

In this prognostic study of adult hospital discharges, hospital readmission risk varied over time and was not captured by a single binary 30-day estimate. These findings suggest that time-structured readmission modeling can characterize when readmission risk occurs, but additional work is needed before such models can reliably guide interventions during specific postdischarge intervals.

## Key Points

**Question:**

Does examining 30-day hospital readmission risk across cumulative horizons and landmark postdischarge intervals reveal clinically relevant differences obscured by a single binary 30-day estimate?

**Findings:**

In this prognostic study of 617 841 discharges, most 30-day readmissions occurred within 14 days after discharge. Cumulative-horizon models characterized readmission risk through days 7, 14, 21, and 30, whereas landmark interval-specific analyses (days 0-7, 8-14, 15-21, and 22-30) showed acceptable discrimination throughout but modest positive predictive values beyond day 7.

**Meaning:**

In contrast to a single binary 30-day estimate, these findings suggest time-structured readmission modeling characterizes when readmission risk occurs, but additional work is needed before such models can reliably identify patients for specific postdischarge follow-up intervals.

## Teaser

This prognostic study evaluates whether examining 30-day hospital readmission risk across 4 time horizons can characterize when readmission risk occurs and potentially identify patients for specific postdischarge follow-up intervals.

## Introduction

In the US, 30-day hospital readmissions remain a major challenge and are associated with patient burden, adverse outcomes, and increased costs. National estimates suggest that approximately 13.3% of discharged patients are readmitted within 30 days.^1^ Most prior studies, including those using machine learning approaches, conceptualize readmission as a single binary 30-day outcome.^2,3^ However, this approach does not distinguish when risk occurs during the postdischarge period. As a result, it provides limited guidance for aligning transitional care strategies with periods when risk is greatest and when it can be most effectively targeted.^4,5^

This limitation has important clinical implications. Transitional care resources are finite, and only a subset of readmissions may be preventable.^6,7^ Clinicians and health systems must therefore determine not only which patients are at highest risk, but also when that risk is most concentrated. Early and later readmissions may differ in how well risk can be identified and in responsiveness to intervention, yet they are typically considered together within a single 30-day framework. A time-structured understanding of readmission risk may better inform how and when to deploy postdischarge care.

To address this knowledge gap, we characterized the timing of readmission risk and its implications for clinical utility. Rather than treating readmission as a single 30-day outcome, we examined first readmission across multiple postdischarge time horizons while accounting for death as a competing event. We estimated readmission risk at 7, 14, 21, and 30 days after discharge and evaluated, using landmark interval-specific analyses, whether time-structured readmission modeling could identify patients for specific postdischarge follow-up intervals.

## Methods

### Study Design and Data Sources

We conducted a prognostic study to examine postdischarge readmission risk across time horizons and its clinical relevance. The development cohort was derived from Epic electronic health record (EHR) data from all 19 Mayo Clinic hospitals in Minnesota, Arizona, Florida, and Wisconsin between July 2017 and March 2024. The external evaluation cohort was the publicly available Medical Information Mart for Intensive Care IV (MIMIC-IV), which included hospitalizations from 2008 to 2019.^8,9^ This study followed Transparent Reporting of a Multivariable Prediction Model for Individual Prognosis or Diagnosis (TRIPOD+AI) guidelines for reporting prediction model studies.^10^ Analyses were conducted from November 2025 through June 2026. The Mayo Clinic institutional review board determined the study to be exempt and granted a waiver of informed consent. MIMIC-IV is a deidentified public database for which investigators complete required data use training and agreements.

### Study Population

The unit of analysis was the index hospital discharge. Eligible discharges were inpatient encounters with a recorded discharge date and time among patients discharged alive. We excluded patients younger than 18 years, those with missing or unknown birth dates, and those who did not provide required research authorization. Eligible encounters included inpatient, transitional care, and hospice admissions. The final development cohort included 617 841 discharges from 341 824 patients. We used MIMIC-IV for external evaluation of the analytical framework (eMethods in Supplement 1). The flow diagram summarizes cohort selection (eFigure 1 in Supplement 1). Because predictors differed between cohorts, models were developed separately using a consistent analytic framework across time horizons.

### Primary Outcome

The outcome was first hospital readmission after discharge. Landmark interval-specific readmission risk was evaluated through days 0 to 7, 8 to 14, 15 to 21, and 22 to 30 among patients who were alive and readmission-free at the start of each interval. Cumulative readmission risk was evaluated through 7, 14, 21, and 30 days after discharge. Death was treated as a competing event.^11^

### Predictors

Candidate predictors were structured EHR variables available at or before discharge, including demographics, social determinants of health, prior utilization, comorbidity indices, features of the index hospitalization, vital signs, laboratory summaries, and discharge medications. A total of 277 candidate predictors were available for analysis; predictor definitions, feature engineering, and hyperparameters are provided in the eMethods in Supplement 1. To assess the contribution of prespecified clinical domains, we performed staged domain ablation analyses within each cohort using the same grouped validation framework. Race and ethnicity were obtained from a self-reported structured EHR field and were defined by the source EHR rather than by the investigators. Race and ethnicity were assessed to describe the cohort and to account for differences in social context, access, and care patterns relevant to postdischarge outcomes.

### Modeling Approach

We developed a discrete-time competing-risk model using gradient-boosted decision trees (XGBoost),^12^ and applied the same analytic framework separately in the development and external cohorts.^11,12^ Analyses used 5-fold cross-validation grouped by patient identifier. Models were fit for cumulative-horizon and landmark analyses. During the study period, patients could contribute multiple discharges; analyses accounted for clustering of discharges within patients to avoid information leakage. Additional analyses assessed temporal and geographic consistency within the development cohort (eMethods in Supplement 1). Performance estimates were based on out-of-fold predictions.

### Clinical Utility and Operational Analyses

Clinical utility was assessed using landmark interval-specific predicted risks to evaluate timing-specific follow-up decisions and cumulative-horizon predicted risks to characterize overall readmission risk. Net benefit was computed across a range of threshold probabilities. For landmark decision-curve analyses, model-based strategies were compared with a treat-none reference strategy.^13,14^ To support operational planning, we evaluated capacity-constrained targeting strategies in which an intervention was offered to the top 5%, 10%, or 20% of discharges ranked by predicted risk. For each strategy, we reported positive predictive value (PPV), the proportion of readmission events captured, and the corresponding risk threshold at each intervention capacity. In landmark analyses, PPV was defined as the proportion of selected patients who experienced readmission during the corresponding interval.

To connect targeting decisions with potential clinical outcomes, we calculated the number needed to treat (NNT) assuming a 20% relative risk reduction.^15,16^ These estimates are illustrative, reflect a hypothetical intervention effect, and should not be interpreted as causal effects.

### Conventional Binary 30-Day Readmission Model

To benchmark against conventional approaches, we fit a binary XGBoost model to predict first readmission within 30 days, defined as occurring before death. The model used the same predictors and validation approach as the primary analyses. We compared performance of the binary model with the cumulative time-structured framework to assess whether modeling readmission across multiple time horizons provided information beyond a single binary 30-day estimate.

### Statistical Analysis

Model performance was assessed using 5-fold cross-validation stratified by readmission status and grouped by patient. Discrimination was evaluated using area under the receiver operating characteristic curve (AUROC) and the area under the precision-recall curve (AUPRC). Calibration was assessed using observed and predicted risk and calibration slope. Overall accuracy was summarized using the Brier score. Ninety-five percent CIs for performance and clinical utility metrics were computed using patient-level cluster bootstrap resampling (1000 iterations), in which patient clusters were sampled with replacement to account for correlation among multiple discharges per patient. Missing numeric values were retained and supplemented with missingness indicators, and sensitivity analyses evaluated alternative missing data handling strategies (eMethods in Supplement 1). Clinical utility was assessed using decision curve analysis and capacity-constrained analyses. For timing-specific clinical utility analyses, PPV and NNT were calculated using landmark interval-specific predictions. The NNT was estimated as NNT = 1 / (PPV × RRR), where PPV is calculated among model-selected patients and RRR is the assumed relative risk reduction (20%) based on meta-analytic estimates of the association between outpatient follow-up and 30-day readmissions.^15,16^ Statistical uncertainty was summarized using 2-sided 95% CIs. No formal hypothesis tests were performed, and statistical significance was not inferred from whether CIs excluded a null value because the analyses focused on prediction performance, calibration, and clinical utility rather than causal effect estimation. The 95% CIs were used to describe the precision of estimates. Analyses were conducted in Python version 3.10.16 (Python Software Foundation).

## Results

### Study Cohort and Hospital Readmission Patterns

The development cohort included 617 841 discharges among 341 824 patients (Table 1). The median (IQR) age was 65 (50-76) years, and 315 220 discharges (51.0%) were among female patients. A total of 24 882 discharges (4.0%) were among Black patients, 11 962 (1.9%) were among Asian patients, and 557 836 (90.3%) were among White patients. The overall 30-day readmission rate was 16.3% (95% CI, 16.2%-16.5%). Readmission occurred predominantly early after discharge: 7.6% (95% CI, 7.5%-7.7%) by 7 days, increasing to 11.1% (95% CI, 11.0%-11.2%) by 14 days and 16.3% by 30 days. Overall, 68 797 30-day readmissions (68.2%) occurred within 14 days of discharge, whereas only 15 482 (15.4%) occurred between days 22 and 30, demonstrating that readmission risk was concentrated early after discharge.

**Table 1.** Characteristics of Hospital Discharges by 30-Day Readmission Status and Timing of Readmission in the Development Cohort

Characteristic	Participants, No. (%)						
	Overall (N = 617 841)	30-d Readmission					No 30-d readmission (n = 517 030)
		Total (n = 100 811)	Timing of readmission				
			0-7 d (n = 46 894)	8-14 d (n = 21 903)	15-21 d (n = 16 532)	22-30 d (n = 15 482)	
Age, median (IQR), y	65 (50-76)	67 (54-76)	68 (55-77)	65 (53-75)	65 (52-75)	67 (55-76)	65 (49-75)
Sex or gender							
Female	315 220 (51.0)	46 831 (46.4)	21 794 (46.5)	10 192 (46.5)	7633 (46.2)	7212 (46.6)	268 389 (51.9)
Male	302 596 (49.0)	53 988 (53.5)	25 109 (53.5)	11 710 (53.5)	8900 (53.8)	8269 (53.4)	248 608 (48.1)
Nonbinary	9 (<0.1)	2 (<0.1)	0	0	1 (<0.1)	1 (<0.1)	7 (<0.1)
Unknown	16 (<0.1)	7 (<0.1)	2 (<0.1)	3 (<0.1)	0	2 (<0.1)	9 (<0.1)
Race							
American Indian or Alaska Native	5479 (0.9)	947 (0.9)	371 (0.8)	235 (1.1)	174 (1.1)	167 (1.1)	4532 (0.9)
Asian	11 962 (1.9)	1820 (1.8)	712 (1.5)	470 (2.1)	334 (2.0)	304 (2.0)	10 142 (2.0)
Black or African American	24 882 (4.0)	4294 (4.3)	1722 (3.7)	998 (4.6)	805 (4.9)	769 (5.0)	20 588 (4.0)
Native Hawaiian or other Pacific Islander	810 (0.1)	128 (0.1)	57 (0.1)	31 (0.1)	20 (0.1)	20 (0.1)	682 (0.1)
White	557 836 (90.3)	91 287 (90.6)	42 936 (91.6)	19 665 (89.8)	14 801 (89.5)	13 885 (89.7)	466 549 (90.2)
Not reported or unknown	16 872 (2.7)	2335 (2.3)	1096 (2.3)	504 (2.3)	398 (2.4)	337 (2.2)	14 537 (2.8)
Rurality							
Metropolitan	395 316 (64.0)	63 101 (62.6)	27 136 (57.9)	14 452 (66.0)	11 055 (66.9)	10 458 (67.5)	332 215 (64.3)
Micropolitan	91 765 (14.9)	15 021 (14.9)	7288 (15.5)	3177 (14.5)	2366 (14.3)	2190 (14.1)	76 744 (14.8)
Small town	71 023 (11.5)	12 550 (12.4)	7236 (15.4)	2204 (10.1)	1640 (9.9)	1470 (9.5)	58 473 (11.3)
Rural	56 071 (9.1)	9320 (9.2)	4865 (10.4)	1838 (8.4)	1365 (8.3)	1252 (8.1)	46 751 (9.0)
Not reported or unknown	3666 (0.6)	819 (0.8)	369 (0.8)	232 (1.1)	106 (0.6)	112 (0.7)	2847 (0.6)
Education							
≥College	228 778 (37.0)	34 431 (34.2)	14 803 (31.6)	7974 (36.4)	6078 (36.8)	5576 (36.0)	194 347 (37.6)
Moderately educated	21 341 (3.5)	3863 (3.8)	1825 (3.9)	830 (3.8)	593 (3.6)	615 (4.0)	17 478 (3.4)
Some college, no degree	54 142 (8.8)	8897 (8.8)	3774 (8.0)	2045 (9.3)	1615 (9.8)	1463 (9.4)	45 245 (8.8)
Rarely educated	119 270 (19.3)	21 728 (21.6)	10 322 (22.0)	4609 (21.0)	3499 (21.2)	3298 (21.3)	97 542 (18.9)
Not reported or unknown	194 310 (31.4)	31 892 (31.6)	16 170 (34.5)	6445 (29.4)	4747 (28.7)	4530 (29.3)	162 418 (31.4)
Employment							
Full-time, part-time, or self-employed	194 479 (31.5)	22 795 (22.6)	10 209 (21.8)	5256 (24.0)	4086 (24.7)	3244 (21.0)	171 684 (33.2)
Retired	301 888 (48.9)	54 410 (54.0)	26 205 (55.9)	11 346 (51.8)	8378 (50.7)	8481 (54.8)	247 478 (47.9)
Disabled	48 707 (7.9)	11 924 (11.8)	5271 (11.2)	2613 (11.9)	2040 (12.3)	2000 (12.9)	36 783 (7.1)
Not employed	69 417 (11.2)	11 423 (11.3)	5065 (10.8)	2641 (12.1)	1988 (12.0)	1729 (11.2)	57 994 (11.2)
Not reported or unknown	3350 (0.5)	259 (0.3)	144 (0.3)	47 (0.2)	40 (0.2)	28 (0.2)	3091 (0.6)
Length of stay, median (IQR), d	3.4 (2.2-5.9)	4.3 (2.7-7.4)	4.3 (2.6-7.9)	4.3 (2.8-7.3)	4.2 (2.8-7.0)	4.1 (2.8-6.9)	3.3 (2.1-5.6)
Death before 30-d readmission	23 822 (3.9)	7226 (7.2)	4744 (10.1)	1529 (7.0)	710 (4.3)	243 (1.6)	16 596 (3.2)

### Time-Structured Readmission Estimates Across Postdischarge Horizons

In the development cohort, we evaluated models using sequential augmentation of prespecified clinical domains, including hospitalization course, vital signs, laboratory results, and medications (eTable 1 in Supplement 1). Inclusion of additional domains was associated with incremental gains in discrimination (AUROC, 0.717 in the base model to 0.790 in the full model) (eTable 2 in Supplement 1). Subsequent analyses are based on the full model. Discrimination was similar across time horizons, with AUROC of 0.812 (95% CI, 0.810-0.814) at 7 days and 0.796 (95% CI, 0.794-0.797) at 30 days, while AUPRC was 0.483 (95% CI, 0.478-0.489) at 7 days and 0.515 (95% CI, 0.511-0.520) at 30 days. The model was well calibrated across time horizons, with predicted risk closely matching observed incidence (Table 2). These cumulative-horizon analyses characterized overall readmission risk across the 30-day postdischarge period but did not directly evaluate timing-specific follow-up decisions.

**Table 2.** Cumulative Model Performance Across Postdischarge Horizons

Cohort and horizon, days	Readmission rate, % (95% CI)^a^	Predicted risk, % (95% CI)^b^	AUPRC (95% CI)^c^	AUROC (95% CI)	Brier score (95% CI)^d^	AUPRC/readmission rate^c^	Calibration slope (95% CI)
Development							
7	7.6 (7.5-7.7)	7.4 (7.3-7.4)	0.483 (0.478-0.489)	0.812 (0.810-0.814)	0.051 (0.050-0.051)	6.36	1.004 (0.995-1.013)
14	11.1 (11.0-11.2)	11.0 (10.9-11.0)	0.470 (0.466-0.474)	0.797 (0.794-0.798)	0.077 (0.076-0.078)	4.23	0.992 (0.985-0.999)
21	13.8 (13.7-13.9)	13.8 (13.7-13.9)	0.491 (0.487-0.495)	0.796 (0.794-0.798)	0.093 (0.093–0.094)	3.56	0.989 (0.981-0.997)
30	16.3 (16.2-16.5)	16.5 (16.4-16.5)	0.515 (0.511-0.520)	0.796 (0.794-0.797)	0.107 (0.106–0.108)	3.16	0.985 (0.977-0.992)
External							
7	5.6 (5.5-5.7)	5.4 (5.4-5.5)	0.193 (0.185-0.202)	0.746 (0.740-0.751)	0.049 (0.048–0.050)	3.45	1.015 (0.994-1.036)
14	9.5 (9.4-9.7)	9.4 (9.3-9.5)	0.287 (0.278-0.297)	0.760 (0.756-0.764)	0.078 (0.076–0.079)	3.02	1.013 (0.996-1.030)
21	12.6 (12.4-12.8)	12.6 (12.5-12.8)	0.378 (0.370-0.387)	0.775 (0.771-0.778)	0.094 (0.093–0.096)	3.00	1.013 (0.998-1.026)
30	15.3 (15.0-15.5)	15.6 (15.4-15.7)	0.436 (0.427-0.444)	0.781 (0.778-0.784)	0.108 (0.107–0.109)	2.85	1.002 (0.990-1.014)

Abbreviations: AUPRC, area under the precision-recall curve; AUROC, area under the receiver operating characteristic curve.

^a^ Readmission rate represents cumulative incidence of first readmission at each horizon.

^b^ Predicted risk represents the mean predicted readmission probability from out-of-fold cross-validation predictions. Ninety-five percent CIs were computed using patient-level cluster bootstrap (1000 iterations).

^c^ AUPRC reflects the ability to identify patients who experience readmission within each time horizon.

^d^ Brier score is the mean squared difference between the predicted readmission probability and the observed readmission outcome at each horizon; lower values indicate better overall prediction accuracy.

Findings were broadly consistent across temporal and geographic validation with modest attenuation in the temporal holdout analyses and variation across hospital sites, reflecting context-dependent transportability (eTable 3 in Supplement 1). Landmark interval-specific models showed acceptable discrimination across postdischarge intervals; however, whether these models could support timing-specific follow-up decisions was evaluated using landmark PPV and NNT analyses (Table 3; Figure 2; eTable 3 in Supplement 1).

### Clinical Utility and Operational Analyses

**Figure 1.**
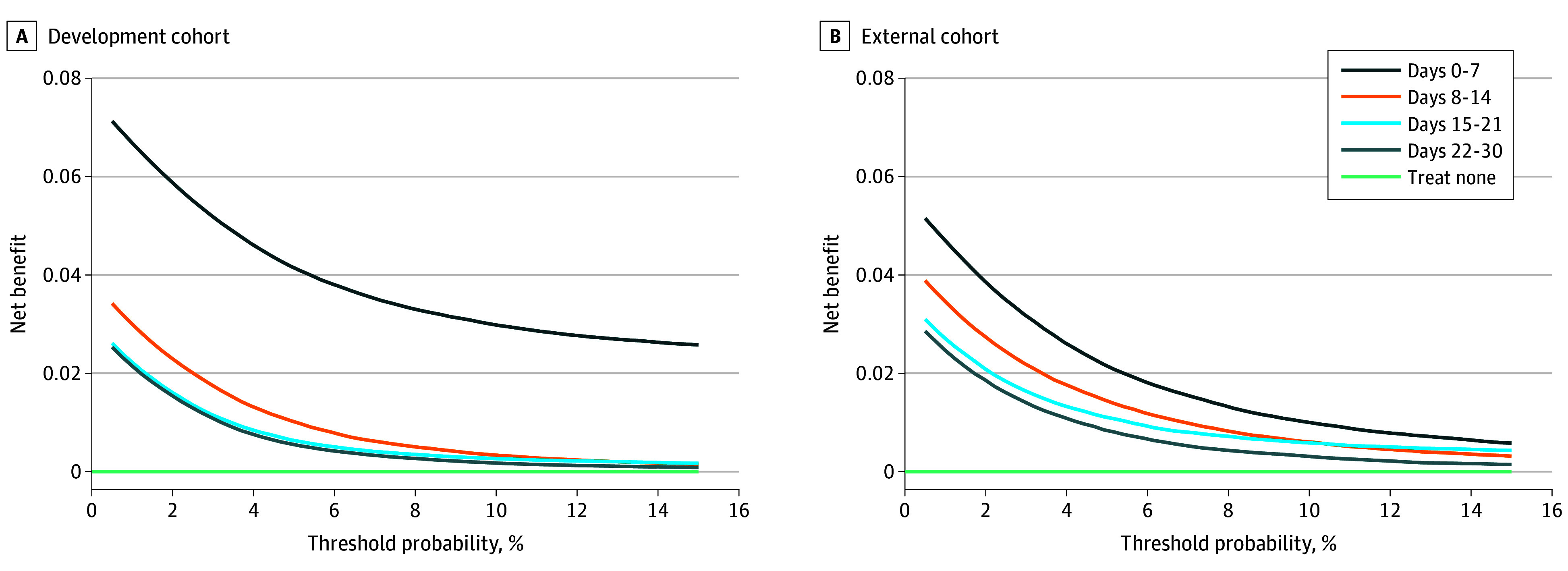
Landmark Decision Curve Analysis Across Postdischarge Intervals Decision-curve analyses for landmark interval-specific readmission predictions during days 0 to 7, 8 to 14, 15 to 21, and 22 to 30 after discharge. Landmark analyses estimated readmission risk during each interval among patients alive and readmission-free at the start of that interval. Higher net benefit indicates greater potential clinical utility of interval-specific risk stratification.

Decision-curve analyses using landmark interval-specific predictions showed positive net benefit compared with treat-none across the displayed threshold probabilities in both the development and external cohorts (Figure 1). Net benefit was greatest for the days 0 to 7 interval and lower for later landmark intervals in both cohorts, consistent with lower interval-specific event rates after the first week.

**Figure 2.**
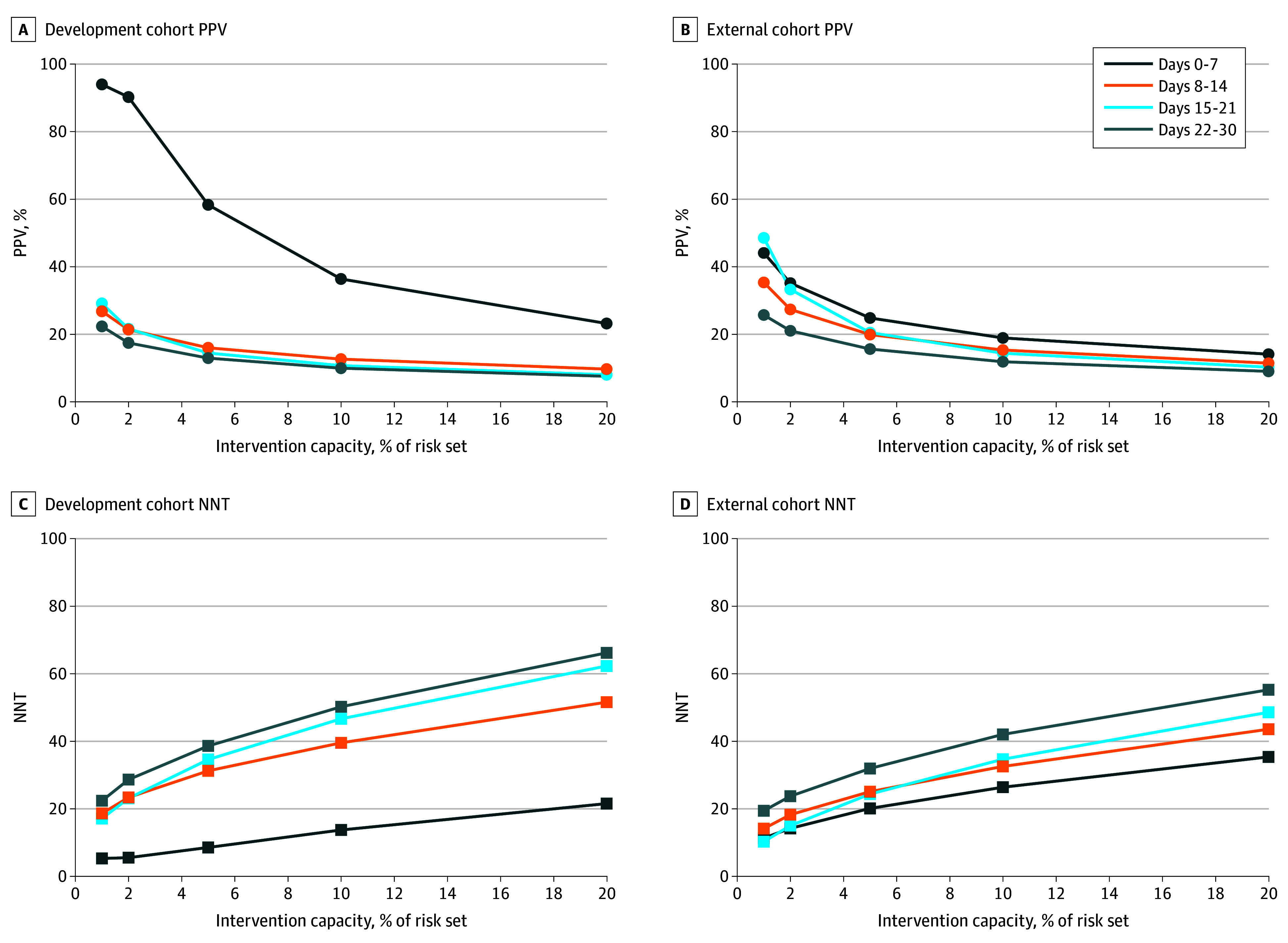
Line Chart Showing Landmark Clinical Utility Across Intervention Capacity Landmark PPV and NNT are shown across intervention capacities for postdischarge intervals 0 to 7, 8 to 14, 15 to 21, and 22 to 30 days. PPV was defined as the proportion of selected patients who experienced readmission during the specified landmark interval among patients alive and readmission-free at the start of that interval. NNT was calculated assuming a 20% relative risk reduction. PPV decreased and NNT increased across later postdischarge intervals, indicating lower timing-specific clinical utility beyond day 7. NNT indicates number needed to treat; PPV, positive predictive value.

In capacity-constrained analyses, cumulative-horizon models showed higher PPV at later horizons because readmission events accumulated over longer follow-up. At 5% intervention capacity in the development cohort, cumulative PPV increased from 58.5% at 7 days to 74.3% at 30 days, corresponding to apparent NNTs of 8.6 and 6.7, respectively. However, when interval-specific risk was evaluated using landmark analyses, PPVs were lower beyond day 7. Landmark PPVs were 58.3% (95% CI, 57.4%-59.1%) for days 0 to 7, 16.0% (95% CI, 15.5%-16.6%) for days 8 to 14, 14.5% (95% CI, 13.9%-15.0%) for days 15 to 21, and 12.9% (95% CI, 12.4% to 13.5%) for days 22 to 30, corresponding to NNTs of 8.6, 31.3, 34.6, and 38.6, respectively, assuming a 20% relative risk reduction (Table 3; Figure 2).

**Table 3.** Landmark and Cumulative Clinical Utility Across Intervention Capacities in the Development Cohort

Cumulative interval, d	Landmark interval, d	Intervention capacity, %^b^	Interval event rate, %^c^	PPV, %		NNT^a^	
				Cumulative^d^	Landmark^e^	Cumulative^d^	Landmark^e^
0-7	0-7	5	7.6	58.5	58.3	8.6	8.6
		10	7.6	36.6	36.4	13.6	13.7
		20	7.6	23.4	23.2	21.4	21.6
0-14	8-14	5	3.9	64.6	16	7.7	31.3
		10	3.9	44.9	12.6	11.1	39.5
		20	3.9	31.4	9.7	15.9	51.6
0-21	15-21	5	3.1	69.8	14.5	7.2	34.6
		10	3.1	51.7	10.7	9.7	46.7
		20	3.1	37.7	8	13.3	62.3
0-30	22-30	5	3	74.3	12.9	6.7	38.6
		10	3	57.2	10	8.7	50.2
		20	3	43.1	7.6	11.6	66.2

Abbreviations: NNT, number needed to treat; PPV, positive predictive value.

^a^ NNT calculations assumed a 20% relative risk reduction.

^b^ Intervention capacity is the proportion of patients selected for intervention based on predicted risk.

^c^ Interval event rate is the observed readmission rate during the specified landmark interval among patients alive and readmission-free at the start of that interval.

^d^ Cumulative PPV and NNT refer to cumulative readmission by the end of the interval.

^e^ Landmark PPV and NNT refer to readmission during the specified interval.

Across intervention capacities, landmark PPVs decreased and NNTs increased as larger proportions of the risk set were selected (Table 3; Figure 2). This pattern was also observed in the external cohort, although absolute PPVs were lower and NNTs were higher than in the development cohort (Figure 2).

Compared with cumulative-horizon estimates, landmark interval-specific PPVs were similar during days 0 to 7 but substantially lower thereafter. These findings demonstrate that overall readmission risk and timing-specific actionability are associated but distinct concepts. Additional cumulative PPV results across intervention capacities and prediction horizons are shown in eFigure 2 in Supplement 1. Landmark readmissions captured across intervention capacities are shown in eFigure 3 in Supplement 1. Cumulative readmissions captured and cumulative NNT estimates across intervention capacities are shown in eFigure 4 in Supplement 1.

### External Evaluation of Analytical Approach

The external cohort included 267 491 discharges among 138 099 patients (eTable 4 in Supplement 1). The median (IQR) age was 62 (45-75) years, and 141 883 discharges (53.0%) were among female patients. A total of 39 455 discharges (14.8%) were among Black patients, 9539 (3.6%) were among Asian patients, and 179 728 (67.2%) were among White patients. The 30-day readmission rate was 15.3% (95% CI, 15.0%-15.5%).

Cumulative model performance in the external cohort was broadly similar to the development cohort, although discrimination was lower at earlier horizons. AUROC was 0.746 (95% CI, 0.740-0.751) at 7 days and 0.781 (95% CI, 0.778-0.784) at 30 days, while AUPRC was 0.193 (95% CI, 0.185-0.202) at 7 days and 0.436 (95% CI, 0.427-0.444) at 30 days. Calibration was adequate, with predicted risk closely matching observed incidence (Table 2).

External landmark interval-specific models showed acceptable discrimination across postdischarge intervals. AUROCs were 0.741 for days 0 to 7, 0.756 for days 8 to 14, 0.782 for days 15 to 21, and 0.766 for days 22 to 30. Patterns of timing-specific clinical utility were similar to those observed in the development cohort (Figure 2).

### Predictor Domain Contributions

Feature-importance analyses showed a consistent structure of predictor domains across the development and external cohorts. Transition-of-care variables, baseline illness burden, and prior utilization contributed the largest signal, whereas physiologic and medication-related variables contributed more modest incremental information (eTable 7 in Supplement 1). Variables included in the external cohort staged ablation domains are listed in eTable 5 in Supplement 1, and sequential augmentation results for the external cohort are shown in eTable 6 in Supplement 1.

### Comparison with Conventional Binary 30-Day Readmission Prediction

As a benchmark, a conventional binary 30-day readmission model using the same predictors achieved equivalent discrimination in the development cohort compared with the 30-day cumulative time-structured model (AUROC, 0.796; 95% CI, 0.794-0.797) for both models. In the external cohort, the binary model showed lower discrimination than the 30-day cumulative time-structured model (AUROC, 0.755; 95% CI, 0.752-0.759 vs 0.781; 95% CI, 0.778-0.784). These findings suggest that conventional binary models capture overall 30-day readmission risk, whereas time-structured models additionally characterize when readmission risk occurs across the postdischarge period. Binary model performance metrics are reported in eTable 8 in Supplement 1.

## Discussion

In this analysis of 617 841 discharges in the development cohort and 267 491 discharges in the external cohort, most 30-day readmissions occurred within the first 14 days after discharge. Cumulative-horizon models characterized how readmission risk accumulated through 7, 14, 21, and 30 days and showed higher apparent enrichment at later horizons. However, landmark interval-specific analyses showed that timing-specific PPVs were modest after day 7. Together, these findings suggest that overall readmission risk and timing-specific actionability are associated but distinct concepts. A single 30-day readmission estimate captures overall risk but obscures when that risk occurs and whether patients can be identified for specific postdischarge follow-up intervals.

Patterns of predictor importance may explain differences in the timing of readmission risk and its clinical utility. Across both cohorts, transition-of-care variables (discharge disposition or location) and baseline illness burden (prior health care utilization or comorbidity) were the dominant predictors, whereas physiologic and medication variables contributed less. These findings suggest that discharge-time models primarily capture underlying vulnerability and care context rather than the dynamic recovery process after hospitalization. This may help explain why cumulative-horizon models effectively characterized overall readmission risk, whereas landmark interval-specific analyses showed more modest PPVs beyond day 7. For clinicians and health systems, the key question is not only which patients are at risk within 30 days, but also when that risk is most actionable. Time-structured approaches may help inform postdischarge planning by making this distinction explicit. The causal implications of these findings should be evaluated in future studies.

The decision-analytic and capacity-constrained findings highlight the distinction between overall readmission risk and timing-specific follow-up decisions. Landmark decision-curve analyses showed a positive net benefit compared with treat-none, supporting the potential value of interval-specific risk stratification. However, cumulative-horizon analyses showed higher apparent enrichment at later horizons because readmission events accumulated over longer follow-up. When the clinically relevant landmark intervals were evaluated, PPVs after day 7 were modest and NNTs were higher. These findings suggest that current discharge-based models may be most useful for identifying patients at high early postdischarge risk, whereas targeting later follow-up windows may require recalculating risk after discharge rather than relying only on information available at discharge. Taken together, these findings suggest that discharge-based models can identify overall readmission risk but may not predict the timing of later readmissions with sufficient precision to guide follow-up scheduling. This limitation could reflect the increasing influence of postdischarge factors that are not fully captured by information available at discharge.

Accounting for the competing risk of death strengthens the clinical interpretation of our findings by yielding risk estimates that more closely reflect patient trajectories in clinical practice. Although a conventional binary 30-day model provided similar overall discrimination, the rolling-horizon approach offers additional insight into when risk occurs; landmark analyses are needed to evaluate whether timing-specific predictions can support follow-up scheduling. These findings build on prior work showing that postdischarge readmission risk changes over time and remains elevated across the weeks following hospitalization, with evolving causes and patterns of identification.^4,5^ However, most readmission prediction tools and quality frameworks continue to rely on a single 30-day risk estimate.^3^ Commonly used risk scores such as the length of stay, acuity of admission, comorbidity, and emergency department visits (LACE) index, originally developed to estimate 30-day readmission risk, provide only moderate discrimination across settings and are not designed to capture how risk evolves over time.^17,18^ These findings may explain the variable effectiveness of readmission interventions. Although systematic reviews have demonstrated modest and inconsistent benefit overall, many interventions rely on risk assessment performed at discharge.^15,19,20^ For later postdischarge intervals, modest landmark PPVs suggest that discharge-time information alone may be insufficient to identify when intervention is most needed. This mismatch between identifying patients at high overall 30-day readmission risk and identifying the interval during which intervention may be most useful may contribute to the limited effectiveness of risk-based readmission interventions.

### Strengths and Limitations

This study has several strengths. It included a large, contemporary cohort spanning multiple years and hospitals across different US regions, with external evaluation in a geographically distinct population. The analysis incorporated a broad set of structured features available at discharge and explicitly accounted for the competing risk of death. Importantly, the study distinguished overall 30-day readmission risk from timing-specific actionability, highlighting the challenge of identifying patients for specific postdischarge follow-up intervals.

This study also has limitations. The analysis was retrospective and limited to structured EHR data available at discharge, which may not fully capture social context, caregiver support, or discharge readiness. Models did not incorporate postdischarge information (eg, outpatient encounters or clinical notes), which may influence readmission risk trajectories. Interval-specific readmission rates after day 7 were low, which limited landmark PPV despite acceptable discrimination. As a result, landmark models may not provide sufficient precision to reliably assign patients to specific later follow-up windows. Readmissions to hospitals outside the source health systems were not captured, which may have led to underascertainment of events. Although externally evaluated in an independent dataset, further validation across diverse health systems is warranted. Decision-curve and scenario-based analyses depend on assumptions about intervention effectiveness and should not be interpreted as estimates of causal treatment benefit.

## Conclusions

In this prognostic study of 617 841 discharges in the development cohort and 267 491 discharges in the external cohort, a rolling-horizon competing-risk approach to readmission prediction demonstrated that both readmission risk and its clinical utility varied over time after discharge. A single 30-day readmission estimate captured overall risk but obscured the distinction between cumulative readmission risk and timing-specific follow-up decisions. Cumulative-horizon models characterized how readmission risk accumulated over 30 days, whereas landmark interval-specific analyses showed modest PPVs after the first week. These findings suggest that time-structured readmission modeling can clarify the difference between overall risk prediction and timing-specific actionability, but current discharge-based models may not yet reliably guide assignment of patients to specific postdischarge follow-up windows.

## Notes

Supplement 1. eMethods. eTable 1. Variables in Staged Ablation Domains for Development Cohort eTable 2. Sequential Augmentation of Clinical Domains in the Development Cohort (30-Day Readmission) eTable 3. Landmark, Temporal, and Geographic Validation Analyses in the Development Cohort eTable 4. Characteristics of Hospital Discharges by Readmission Status and Timing in the External Cohort eTable 5. Variables in Staged Ablation Domains for External Cohort eTable 6. Sequential Augmentation of Clinical Domains in the External Cohort (30-Day Readmission) eTable 7. Clinical Domains Contributing to Readmission Predictability Across Time Horizons eTable 8. Conventional Binary 30-Day Readmission Prediction in Development and External Cohorts eFigure 1. Cohort Selection for Development and External Cohorts eFigure 2. Positive Predictive Value Across Intervention Capacity and Prediction Horizons eFigure 3. Landmark Readmissions Captured Across Intervention Capacity eFigure 4. Cumulative Readmissions Captured and Number Needed to Treat Across Intervention Capacity eReferences.

Supplement 2. Data Sharing Statement
