# Supplementary material for: Timing of 30-Day Hospital Readmission Risk and Its Implications for Clinical Utility
Source: JAMA Netw Open. 2026 Aug 13;9(8):e2628846. doi: 10.1001/jamanetworkopen.2026.28846 (PMC13474041; doi:10.1001/jamanetworkopen.2026.28846)
Supplement: Supplementary file 1 [file jamanetwopen-e2628846-s001.pdf]

Supplemental Online Content

Alkayali MZ, Fu Y, Castro JC, Gill M, Ayanian S, Dugani SB. Timing of 30-day hospital readmission risk and implications for clinical utility. *JAMA Netw Open*. 2026;9(8):e2628846. doi:10.1001/jamanetworkopen.2026.28846

eMethods.

eTable 1. Variables in Staged Ablation Domains for Development Cohort

eTable 2. Sequential Augmentation of Clinical Domains in the Development Cohort (30-Day Readmission)

eTable 3. Landmark, Temporal, and Geographic Validation Analyses in the Development Cohort

eTable 4. Characteristics of Hospital Discharges by Readmission Status and Timing in the External Cohort

eTable 5. Variables in Staged Ablation Domains for External Cohort

eTable 6. Sequential Augmentation of Clinical Domains in the External Cohort (30-Day Readmission)

eTable 7. Clinical Domains Contributing to Readmission Predictability Across Time Horizons

eTable 8. Conventional Binary 30-Day Readmission Prediction in Development and External Cohorts

eFigure 1. Cohort Selection for Development and External Cohorts

eFigure 2. Positive Predictive Value Across Intervention Capacity and Prediction Horizons

eFigure 3. Landmark Readmissions Captured Across Intervention Capacity

eFigure 4. Cumulative Readmissions Captured and Number Needed to Treat Across Intervention Capacity

eReferences.

This supplemental material has been provided by the authors to give readers additional information about their work.

© 2026 Alkayali MZ et al. *JAMA Network Open*.

© 2026 Alkayali MZ et al. *JAMA Network Open*.

eMethods

1. Cohort Construction and Outcome Definitions

Index Discharges

Each row in the analytic dataset corresponds to an index hospital discharge. Discharges were required to have valid admission and discharge timestamps and to represent survival to discharge. Multiple discharges per patient were permitted and were handled using patient-level grouping during analysis.

Postdischarge Events

Two postdischarge events within 30 days were captured: hospital readmission and death. Readmission was defined as the first inpatient admission within 30 days after discharge, and postdischarge death was defined as death within 30 days after discharge. Event type was classified based on the first event within 30 days: first readmission if readmission occurred before or on the same day as death, death as the first event if death occurred before any readmission, and no event if neither occurred. Time to first event was defined as the minimum of time from discharge to readmission, time to death, or administrative censoring at 30 days.

2. Predictor Domains and Preprocessing

Predictor Domains

Predictors were grouped into prespecified domains to support staged ablation and assessment of consistency across cohorts. Predictors were defined using information available at or before hospital discharge. Domains included demographics and social determinants of health (e.g., age, sex, race group, and insurance), prior health care utilization (e.g., prior admissions and emergency department visits), comorbidity (e.g., Elixhauser Comorbidity Index), features of the index hospitalization (e.g., length of stay, ICU utilization, and procedures), summary measures of vital signs and laboratory values (e.g., minimum, maximum), and discharge medications (e.g., therapeutic classes and medication counts). Sex categories were extracted directly from the source EHR as recorded and included female, male, nonbinary, and unknown. Categories were not reclassified by the investigators.

Missingness Handling Variants

Three missing data handling approaches were evaluated

- Variant A (primary): retain missing numeric values (native handling by tree-based models), include missingness indicators, and add laboratory measurement intensity features (counts and fractions).
- Variant B: median imputation for numeric predictors plus missingness indicators.
- Variant C: Variant A plus exclusion of laboratory predictors exceeding a prespecified missingness threshold (e.g., >95% missing).

3. Model Specifications

Binary Model (30-Day Readmission)

We trained a gradient-boosted decision tree model (XGBoost) with a logistic objective to predict first readmission within 30 days. Class weighting was not used. Categorical predictors were handled using XGBoost's native categorical split support after appropriate encoding. Key hyperparameters, aligned with the released code, included:

- n_estimators up to 5000 with early stopping
- learning_rate approximately 0.03 to 0.05
- max_depth approximately 4 to 6
- subsample approximately 0.8
- colsample_bytree approximately 0.8
- reg_lambda approximately 1.0
- tree_method = "hist"
- eval_metric = "logloss"

4. Discrete-Time Rolling-Horizon Competing-Risk Model

Postdischarge follow-up was discretized into successive intervals. Each index discharge contributed person-period records until the first event or administrative censoring. Interval-specific event status among patients still at risk was estimated, and interval-level predictions were accumulated to estimate the cumulative incidence of first readmission by through days 7, 14, 21, and 30. The same analytic framework was applied in each cohort, but models were fit and evaluated within each cohort rather than transporting a single fitted model across datasets.

Within each training fold, an inner grouped split was used for early stopping. Two cause-specific hazard models were fit using gradient-boosted decision trees (XGBoost): one for first readmission and one for death as the first event. For each interval k,

© 2026 Alkayali MZ et al. *JAMA Network Open*.

the hazard h_k was the predicted probability of the event in that interval conditional on being event-free at the start of the interval. The cumulative incidence function (CIF) of first readmission by the end of interval K was computed according to standard competing-risk methods.[1]

$$CIF\_readmit(K) = \Sigma_{k=1..K} S(k-1) \times h\_readmit(k)$$
Where $S(k) = \Pi_{j=1..k} [1 - h\_readmit(j) - h\_death(j)]$

5. Landmark Analysis

To distinguish cumulative risk by horizon from conditional interval-specific risk, we performed landmark analysis in the development cohort. Four landmark intervals were prespecified: days 0−7, 8−14, 15−21, and 22−30 after discharge. For each interval, the risk set included only discharges that remained alive and readmission-free at the start of the interval. Using the same discharge-time predictors and grouped validation framework, we estimated interval-specific first-readmission risk within each interval, treating death before readmission during the interval as the competing event. Landmark predictions were used for timing-specific clinical utility analyses, including positive predictive value, number needed to treat, and decision-curve analyses.

6. Validation Strategy and Performance Metrics

Cross-Validation
All analyses used 5-fold stratified group cross-validation, with patient identifier as the grouping variable. Stratification was based on the primary binary outcome to ensure similar event prevalence across folds. This strategy prevented information leakage by ensuring that discharges from the same patient were not split across training and test folds.

Discrimination, Calibration, and Performance
Discrimination was assessed using the area under the receiver operating characteristic curve (AUROC) and the area under the precision-recall curve (AUPRC). Overall performance was assessed using the Brier score. Calibration was summarized using (1) mean predicted risk, (2) observed event rate, (3) calibration-in-the-large (CITL = logit[observed] − logit[mean predicted]), and (4) calibration slope from logistic calibration regression of the outcome on the logit of predicted risk.

Decile-based calibration tables were constructed by sorting discharges by predicted risk, splitting them into 10 equal-sized groups, and reporting observed event rates and mean predicted risks within each decile.

Because AUPRC is prevalence dependent, cross-horizon comparisons were interpreted alongside the horizon-specific event rate. We additionally report the AUPRC/event-rate ratio descriptively; this ratio was not treated as a primary performance metric.

Temporal Holdout Validation
To assess short-term temporal stability, we trained models on discharges before January 1, 2023, and evaluated them on discharges from January 1, 2023, onward. To prevent leakage across repeated discharges, patients contributing any discharge to the temporal holdout set were excluded from the training set.

Geographic Holdout Validation
To assess internal transportability across care settings, we performed leave-one-region-out validation in the development cohort. For each region, models were trained on all remaining regions and evaluated in the held-out region. These analyses were intended to characterize heterogeneity in discrimination, calibration, and absolute enrichment across geographic contexts rather than to define a single pooled transportability estimate.

7. Decision Curve Analysis

For a given threshold probability p_t, net benefit was calculated as:
$$NB(p_t) = (TP/N) - (FP/N) \times p_t/(1-p_t)$$
where TP and FP denote the numbers of true positive (TP) and false positive (FP) classifications when predicted risk was at or above p_t. For each landmark interval, net benefit was calculated separately among patients alive and readmission-free at the start of that interval. Model-based landmark strategies were compared with a treat-none reference strategy.

8. Capacity Analysis and Number Needed to treat (NNT) Scenarios

For capacity analyses, discharges were ranked by predicted risk and flagged if their risk was in the top X% ($X \in \{5, 10, 20\}$). The implied risk threshold was the (1−X) quantile of predicted risk. For cumulative-horizon analyses, positive predictive value (PPV) was defined as the proportion of selected discharges with readmission by the specified horizon. For landmark interval-specific analyses, PPV was defined as the proportion of selected discharges with readmission during the corresponding interval among

© 2026 Alkayali MZ et al. *JAMA Network Open*.

patients alive and readmission-free at the start of that interval. The proportion of events captured was defined as the proportion of all readmissions during the corresponding horizon or interval that occurred among selected discharges.

Number Needed to Treat Derivation for Model-Selected Populations

Among patients selected by the prediction model at a given intervention capacity, PPV represents the baseline event risk, defined as the probability that a selected patient will experience readmission during the specified horizon or interval. When an intervention is applied to this model-selected group with an assumed relative risk reduction (RRR), the absolute risk reduction (ARR) among treated patients is ARR = PPV × RRR. The number needed to treat is therefore NNT = 1/ARR = 1/(PPV × RRR). This formulation applies the standard NNT = 1/ARR definition within the model-selected population rather than the overall population.[2] The assumed 20% RRR was based on prior meta-analytic estimates of the association between outpatient follow-up and 30-day readmissions.[3]

© 2026 Alkayali MZ et al. *JAMA Network Open*.

eTable 1. Variables in Staged Ablation Domains for Development Cohort

Stage	Variables
**Base model**	patient_class_name; region; discharge_disposition_name; patient_zip_code; age_at_admission; patient_sex; admission_day_type; admission_time_of_day; race_group; ethnic_group; language_group; marital_group; employment_group; education_group; rurality; weight; most_recent_BMI_for_visit; is_member_of_tobacco_registry; age_adj_ECI; ECI; LACE+ score
**Hospital course**	length_of_stay; total_npo_duration_hour; diet_types_count; npo_diet; dialysis_required; total_vent_mins; ventilation_required; O_2_severity; diet_order_count_normalized
**Vital signs**	discharge_dbp_count; discharge_dbp_first; discharge_dbp_last; discharge_dbp_max; discharge_dbp_mean; discharge_dbp_min; discharge_hr_count; discharge_hr_first; discharge_hr_last; discharge_hr_max; discharge_hr_mean; discharge_hr_min; discharge_map_count; discharge_map_first; discharge_map_last; discharge_map_max; discharge_map_mean; discharge_map_min; discharge_rr_count; discharge_rr_first; discharge_rr_last; discharge_rr_max; discharge_rr_mean; discharge_rr_min; discharge_sbp_count; discharge_sbp_first; discharge_sbp_last; discharge_sbp_max; discharge_sbp_mean; discharge_sbp_min; discharge_spo2_count; discharge_spo2_first; discharge_spo2_last; discharge_spo2_max; discharge_spo2_mean; discharge_spo2_min; discharge_temp_count; discharge_temp_first; discharge_temp_last; discharge_temp_max; discharge_temp_mean; discharge_temp_min; hr_count_normalized; hr_first; hr_last; hr_max; hr_mean; hr_min; sbp_count_normalized; sbp_first; sbp_last; sbp_max; sbp_mean; sbp_min
**Laboratory results**	alb_count_normalized; alb_first; alb_last; alb_max; alb_mean; alb_min; alt_count_normalized; alt_first; alt_last; alt_max; alt_mean; alt_min; ast_count_normalized; ast_first; ast_last; ast_max; ast_mean; ast_min; bicarb_count_normalized; bicarb_first; bicarb_last; bicarb_max; bicarb_mean; bicarb_min; bili_count_normalized; bili_first; bili_last; bili_max; bili_mean; bili_min; bun_count_normalized; bun_first; bun_last; bun_max; bun_mean; bun_min; ca_count_normalized; ca_first; ca_last; ca_max; ca_mean; ca_min; ck_count_normalized; ck_first; ck_last; ck_max; ck_mean; ck_min; crp_count_normalized; crp_first; crp_last; crp_max; crp_mean; crp_min; crt_count_normalized; crt_first; crt_last; crt_max; crt_mean; crt_min; esr_count_normalized; esr_first; esr_last; esr_max; esr_mean; esr_min; hba1_count_normalized; hba1_first; hba1_last; hba1_max; hba1_mean; hba1_min; hct_count; hct_first; hct_last; hct_max; hct_mean; hct_min; hgb_count_normalized; hgb_first; hgb_last; hgb_max; hgb_mean; hgb_min; k_count_normalized; k_first; k_last; k_max; k_mean; k_min; lact_count_normalized; lact_first; lact_last; lact_max; lact_mean; lact_min; mcv_count_normalized; mcv_first; mcv_last; mcv_max; mcv_mean; mcv_min; mg_count_normalized; mg_first; mg_last; mg_max; mg_mean; mg_min; na_count_normalized; na_first; na_last; na_max; na_mean; na_min; pho_count_normalized; pho_first; pho_last; pho_max; pho_mean; pho_min; plt_count_normalized; plt_first; plt_last; plt_max; plt_mean; plt_min; pt_count_normalized; pt_first; pt_last; pt_max; pt_mean; pt_min; wbc_count_normalized; wbc_first; wbc_last; wbc_max; wbc_mean; wbc_min
**Medications**	discharge_allergy_respiratory_cold_preparations; discharge_analgesics_and_antiarthritics; discharge_antibiotics; discharge_anticoagulants; discharge_antifungals; discharge_antihyperglycemics; discharge_antineoplastics; discharge_antiparasitics; discharge_antiplatelet_agents; discharge_antivirals; discharge_biologicals_and_immunosuppressants; discharge_cardiovascular_cardiac_autonomic_drugs_and_diuretics; discharge_central_nervous_system_drugs_and_anti_parkinsonism_drugs; discharge_colonystimulating_factors_and_hematopoietic_growth_factors; discharge_gastrointestinal_medications; discharge_muscle_relaxants; discharge_other_antiinfectives; discharge_other_endocrine_therapies; discharge_psychotherapeutic_agents; discharge_sedative_hypnotics; discharge_smoking_deterrents; discharge_vitamins_and_prenatal_vitamins_and_herbals; home_allergy_respiratory_cold_preparations; home_analgesics_and_antiarthritics; home_antibiotics; home_anticoagulants; home_antifungals; home_antihyperglycemics; home_antineoplastics; home_antiparasitics; home_antiplatelet_agents; home_antivirals; home_biologicals_and_immunosuppressants; home_cardiovascular_cardiac_autonomic_drugs_and_diuretics; home_central_nervous_system_drugs_and_anti_parkinsonism_drugs; home_colonystimulating_factors_and_hematopoietic_growth_factors; home_gastrointestinal_medications; home_muscle_relaxants; home_other_antiinfectives; home_other_endocrine_therapies; home_psychotherapeutic_agents; home_sedative_hypnotics; home_smoking_deterrents; home_vitamins_and_prenatal_vitamins_and_herbals;

© 2026 Alkayali MZ et al. *JAMA Network Open*.

	mar_allergy_respiratory_cold_preparations; mar_analgesics_and_antiarthritics; mar_antibiotics; mar_anticoagulants; mar_antifungals; mar_antihyperglycemics; mar_antineoplastics; mar_antiparasitics; mar_antiplatelet_agents; mar_antivirals; mar_biologicals_and_immunosuppressants; mar_cardiovascular_cardiac_autonomic_drugs_and_diuretics; mar_central_nervous_system_drugs_and_anti_parkinsonism_drugs; mar_colonystimulating_factors_and_hematopoietic_growth_factors; mar_gastrointestinal_medications; mar_muscle_relaxants; mar_other_antiinfectives; mar_other_endocrine_therapies; mar_psychotherapeutic_agents; mar_sedative_hypnotics; mar_smoking_deterrents; mar_vitamins_and_prenatal_vitamins_and_herbals

Abbreviations: BMI, body mass index; DBP, diastolic blood pressure; ECI, Elixhauser Comorbidity Index; HR, heart rate; ICU, intensive care unit; MAR, medication administration record; MAP, mean arterial pressure; NPO, nothing by mouth; O_2, oxygen; SBP, systolic blood pressure; SpO_2, peripheral oxygen saturation.

© 2026 Alkayali MZ et al. *JAMA Network Open*.

eTable 2. Sequential Augmentation of Clinical Domains in the Development Cohort (30-Day Readmission)

Stage	Variables (N)	AUROC	Δ AUROC	AUPRC	Δ AUPRC	Brier score	Calibration slope
Base model (baseline and discharge information)	26	0.717	Reference	0.321	Reference	0.126	0.998
+ Hospital course	37	0.745	+0.029	0.390	+0.069	0.121	1.001
+ Hospital course + vital signs	85	0.759	+0.014	0.451	+0.061	0.115	0.972
+ Hospital course + vital signs + laboratory results	217	0.774	+0.015	0.471	+0.020	0.113	0.981
+ Hospital course + vital signs + laboratory results + medications	236	0.790	+0.016	0.520	+0.049	0.108	0.993

Abbreviations: AUPRC, area under the precision-recall curve; AUROC, area under the receiver operating characteristic curve.
Δ values represent changes relative to the preceding stage.

© 2026 Alkayali MZ et al. *JAMA Network Open*.

eTable 3. Landmark, Temporal, and Geographic Validation Analyses in the Development Cohort

Validation stratum	Window, d	N	Readmission rate, %	Mean predicted risk, %	AUROC	AUPRC	AUPRC/ Readmission rate	Brier score	Calibration slope
**A. Landmark competing-risk analyses**									
Development cohort	0−7	617,841	7.6	7.4	0.807	0.480	6.33	0.051	1.023
	8−14	563,253	3.9	3.8	0.743	0.129	3.31	0.036	1.024
	15−21	537,625	3.1	3.0	0.755	0.130	4.23	0.028	1.014
	22−30	518,430	3.0	2.9	0.751	0.105	3.51	0.028	1.008
**B. Temporal holdout validation**									
Held-out discharges from Jan 1, 2023 onward	7	70,757	6.3	6.4	0.787	0.419	6.67	0.045	1.063
	14	70,757	9.5	9.9	0.773	0.411	4.34	0.069	1.017
	21	70,757	11.7	12.5	0.775	0.437	3.73	0.084	1.024
	30	70,757	13.8	15.0	0.776	0.459	3.33	0.096	1.019
**C. Leave-one-region-out geographic holdout validation**									
Site 1	7	221,766	5.2	5.0	0.713	0.122	2.34	0.050	0.663
	14	221,766	8.6	8.2	0.727	0.200	2.32	0.076	0.753
	21	221,766	11.2	10.8	0.738	0.263	2.35	0.092	0.807
	30	221,766	13.4	13.3	0.745	0.313	2.34	0.105	0.831
Site 2	7	85,838	5.5	7.0	0.696	0.115	2.10	0.052	0.782
	14	85,838	9.5	11.4	0.711	0.201	2.10	0.083	0.836
	21	85,838	12.7	14.8	0.727	0.277	2.18	0.103	0.889
	30	85,838	15.6	18.0	0.736	0.340	2.18	0.120	0.903
Site 3	7	15,529	3.6	4.8	0.841	0.391	10.76	0.027	0.969
	14	15,529	4.8	6.8	0.823	0.361	7.49	0.037	0.995
	21	15,529	5.6	8.4	0.815	0.355	6.36	0.044	1.008
	30	15,529	6.4	10.0	0.811	0.356	5.57	0.051	1.022
Site 4	7	87,736	5.7	6.2	0.686	0.117	2.06	0.053	0.751
	14	87,736	10.0	10.4	0.708	0.208	2.08	0.086	0.855
	21	87,736	13.4	13.6	0.724	0.288	2.15	0.107	0.916
	30	87,736	16.5	16.6	0.731	0.351	2.12	0.124	0.936
Site 5	7	23,314	6.4	7.6	0.850	0.541	8.39	0.040	0.976
	14	23,314	8.5	10.5	0.823	0.493	5.84	0.057	0.989
	21	23,314	10.0	12.9	0.814	0.485	4.84	0.069	1.002

© 2026 Alkayali MZ et al. *JAMA Network Open*.

	30	23,314	11.6	15.2	0.803	0.477	4.11	0.081	0.995
Site 6	7	39,132	6.8	7.3	0.865	0.550	8.15	0.040	0.938
	14	39,132	8.4	9.6	0.843	0.514	6.10	0.054	0.960
	21	39,132	9.6	11.5	0.834	0.504	5.22	0.063	0.976
	30	39,132	11.0	13.3	0.825	0.499	4.53	0.074	0.984
Site 7	7	65,032	10.8	10.0	0.906	0.755	6.97	0.042	1.305
	14	65,032	13.2	12.8	0.875	0.703	5.33	0.061	1.299
	21	65,032	14.9	15.0	0.860	0.681	4.58	0.074	1.283
	30	65,032	16.7	17.1	0.849	0.670	4.01	0.086	1.271

Randomly assigned site numbers refer to Mayo Clinic hospitals in Rochester, Florida, Arizona, and regions within Mayo Clinic Health System (southeast Minnesota, southwest Minnesota, northwest Wisconsin, southwest Wisconsin).
Temporal holdout analyses showed preserved performance over time. However, leave-one-region-out validation revealed meaningful within-system heterogeneity, indicating context-dependent transportability.
Abbreviations: AUPRC, area under the precision-recall curve; AUROC, area under the receiver operating characteristic curve.

© 2026 Alkayali MZ et al. *JAMA Network Open*.

eTable 4. Characteristics of Hospital Discharges by Readmission Status and Timing in the External Cohort

Characteristic	Overall (N=267,491)	30-Day Readmission					No 30-Day Readmission (N=226,466)
		Total (N=41,025)	Timing of Readmission Within 30 days				
			0−7 d (N=15,107)	8−14 d (N=10,494)	15−21 d (N=8,145)	22−30 d (N=7,279)	
**Age, years**	62 (45−75)	62 (49−75)	62 (47−75)	63 (50−75)	62 (50−74)	64 (51−75)	62 (44−75)
**Female**	141,883 (53.0)	19,969 (48.7)	7,393 (48.9)	5,048 (48.1)	3,989 (49.0)	3,539 (48.6)	121,914 (53.8)
**Race**							
Asian	9,539 (3.6)	1,316 (3.2)	517 (3.4)	359 (3.4)	219 (2.7)	221 (3.0)	8,223 (3.6)
Black	39,455 (14.8)	6,475 (15.8)	2,482 (16.4)	1,557 (14.8)	1,216 (14.9)	1,220 (16.8)	32,980 (14.6)
Hispanic or Latino	14,427 (5.4)	2,160 (5.3)	769 (5.1)	533 (5.1)	449 (5.5)	409 (5.6)	12,267 (5.4)
White	179,728 (67.2)	28,694 (69.9)	10,424 (69.0)	7,482 (71.3)	5,739 (70.5)	5,049 (69.4)	151,034 (66.7)
Other	11,653 (4.4)	1,652 (4.0)	583 (3.9)	414 (3.9)	380 (4.7)	275 (3.8)	10,001 (4.4)
Unknown or declined	12,689 (4.7)	728 (1.8)	332 (2.2)	149 (1.4)	142 (1.7)	105 (1.4)	11,961 (5.3)
**Marital status**							
Divorced	18,371 (6.9)	3,176 (7.7)	1,174 (7.8)	781 (7.4)	650 (8.0)	571 (7.8)	15,195 (6.7)
Married	117,813 (44.0)	18,099 (44.1)	6,424 (42.5)	4,771 (45.5)	3,703 (45.5)	3,201 (44.0)	99,714 (44.0)
Single	91,759 (34.3)	14,402 (35.1)	5,461 (36.1)	3,627 (34.6)	2,766 (34.0)	2,548 (35.0)	77,357 (34.2)
Widowed	30,757 (11.5)	4,925 (12.0)	1,792 (11.9)	1,252 (11.9)	971 (11.9)	910 (12.5)	25,832 (11.4)
Unknown	8,791 (3.3)	423 (1.0)	256 (1.7)	63 (0.6)	55 (0.7)	49 (0.7)	8,368 (3.7)
**Length of stay, days**	4.0 (2.2−7.0)	4.7 (2.8−8.0)	4.6 (2.5−8.2)	4.9 (2.8−8.1)	4.8 (2.8−7.7)	4.6 (2.7−7.8)	3.9 (2.2−6.9)

Data reported as frequency (%) or median (interquartile range)

© 2026 Alkayali MZ et al. *JAMA Network Open*.

eTable 5. Variables in Staged Ablation Domains for External Cohort

Stage	Variables
**Base model**	age; los_days; sex/gender; race; insurance; language; marital_status; admission_type; admission_location; discharge_location; anchor_year_group; admission_day_type; admission_time_of_day; n_prior_admissions_all; n_prior_admissions_365d; n_prior_admissions_30d; any charlson* field and any *_comorbidity_index field joined from the derived charlson table when available
**ICU**	any_icu; n_icu_stays; icu_los_days
**ED vital signs**	ed_triage_temperature; ed_triage_heartrate; ed_triage_resprate; ed_triage_o2sat; ed_triage_sbp; ed_triage_dbp; ed_triage_pain; ed_triage_acuity; ed_vitalsign_n
**Laboratory results**	For each lab stem {na, k, cl, bicarb, bun, crt, glucose, wbc, hgb, hct, plt, lact, ast, alt, bili, alb, mg, pho, ca, inr, pt, mcv, hba1, crp, esr, ck}, the following summaries are created: _min, _max, _mean, _first, _last, _count, _count_normalized.
**Medications**	For each medication class stem {Allergy_respiratory_cold_preparations, analgesics_and_antiarthritics, central_nervous_system_drugs_and_anti_parkinsonism_drugs, other_endocrine_therapies, antibiotics, anticoagulants, Other_antiinfectives, antifungals, antihyperglycemics, biologicals_and_immunosuppressants, antineoplastics, antiparasitics, antiplatelet_agents, antivirals, cardiovascular_cardiac_autonomic_drugs_and_diuretics, colonystimulating_factors_and_hematopoietic_growth_factors, gastrointestinal_medications, vitamins_and_prenatal_vitamins_and_herbals, muscle_relaxants, psychotherapeutic_agents, sedative_hypnotics, Smoking_deterrents}, the pipeline generates Home_, MAR_, and Discharge_ versions.
**Other**	discharge_sbp_min/max/mean/first/last/count; discharge_dbp_min/max/mean/first/last/count; discharge_map_min/max/mean/first/last/count; discharge_hr_min/max/mean/first/last/count; discharge_rr_min/max/mean/first/last/count; discharge_spo2_min/max/mean/first/last/count; discharge_temp_min/max/mean/first/last/count; sbp_min/max/mean/first/last/count_normalized; hr_min/max/mean/first/last/count_normalized; any remaining modeled columns that do not match the earlier naming rules.

Abbreviations: ED, emergency department; ICU, intensive care unit; MAR, medication administration record.
Variables are listed as used in the analytic dataset.

© 2026 Alkayali MZ et al. *JAMA Network Open*.

eTable 6. Sequential Augmentation of Clinical Domains in the External Cohort (30-Day Readmission)

Stage	Variables (N)	AUROC	ΔAUROC	AUPRC	ΔAUPRC	Brier score	Calibration slope
Base model (baseline and discharge information)	16	0.750	Reference	0.371	Reference	0.114	1.035
+ ICU course	19	0.752	+0.002	0.373	+0.002	0.114	1.035
+ ICU course + ED vital signs	28	0.744	-0.008	0.361	-0.011	0.115	1.100
+ ICU course + ED vital signs + laboratory results	196	0.764	+0.020	0.406	+0.044	0.111	0.940
+ ICU course + ED vital signs + laboratory results + medications	260	0.773	+0.009	0.423	+0.018	0.110	1.002
+ ICU course + ED vital signs + laboratory results + medications + other	314	0.756	-0.017	0.355	-0.068	0.117	0.626

Δ values represent changes relative to the preceding stage. Each stage reflects cumulative inclusion of the listed domains. The final stage, which included additional non-domain variables, did not improve discrimination or precision relative to prior stages.

Abbreviations: AUPRC, area under the precision-recall curve; AUROC, area under the receiver operating characteristic curve; ED, emergency department; ICU, intensive care unit.

© 2026 Alkayali MZ et al. *JAMA Network Open*.

eTable 7. Clinical Domains Contributing to Readmission Predictability Across Time Horizons

Clinical domain	Development cohort	External cohort	Clinical interpretation
Transition-of-care (discharge disposition/location)	Discharge disposition was the dominant predictor across all time horizons, with consistent importance from 7 to 30 days (mean ΔAUROC ~0.034).	Discharge location was the leading predictor in both 30-day and rolling-horizon analyses, with strongest influence at earlier time points (mean ΔAUROC ~0.036)	Transition-of-care context is the most consistent and transferable determinant of readmission risk
Baseline illness burden and prior utilization	Comorbidity burden increased in importance with longer prediction horizons (mean ΔAUROC ~0.013).	Prior healthcare utilization showed increasing importance from early to later time windows (mean ΔAUROC ~0.030).	Underlying disease burden becomes more informative at longer time horizons
Encounter and system context (site, time, region)	Regional variation contributed consistently across time horizons (mean ΔAUROC ~0.011).	Admission location and calendar time contributed modest but consistent signal (mean ΔAUROC ~0.007–0.010).	System-level context captures practice patterns and case-mix that influence readmission risk
Markers of complex illness (e.g., oncology-related therapies)	Medication-based proxies for complex illness showed consistent but secondary importance (mean ΔAUROC ~0.002).	Similar medication proxies ranked among secondary predictors (mean ΔAUROC ~0.003–0.004).	Treatment-related variables likely reflect illness complexity rather than causal drivers
Hospital course and physiologic intensity	Markers of inpatient monitoring and physiologic instability contributed modest incremental signal (mean ΔAUROC ~0.002).	Length of stay and laboratory extremes showed similar modest contributions (mean ΔAUROC ~0.001–0.002).	Acute illness severity contributes to risk but less than transition-of-care and baseline-burden

Abbreviation: AUROC, area under the receiver operating characteristic curve.
Clinical domains reflect grouped variables contributing to readmission predictability. Patterns are summarized qualitatively to emphasize consistency across time horizons and cohorts.
Permutation importance is summarized as the mean reduction in out-of-fold AUROC after feature shuffling. These results are presented to characterize the structure of predictability rather than to emphasize individual variables

© 2026 Alkayali MZ et al. *JAMA Network Open*.

eTable 8. Conventional Binary 30-Day Readmission Prediction in Development and External Cohorts

Cohort	AUPRC	AUROC	Brier score
Development	0.517 (0.513–0.521)	0.796 (0.794–0.797)	0.107 (0.106–0.108)
External	0.355 (0.347–0.362)	0.755 (0.752–0.759)	0.118 (0.116–0.119)

Values are presented as estimates with 95% confidence intervals (CIs).
AUPRC reflects the ability to identify patients who experience readmission within 30 days.
Abbreviations: AUPRC, area under the precision-recall curve; AUROC, area under the receiver operating characteristic curve.

© 2026 Alkayali MZ et al. *JAMA Network Open*.

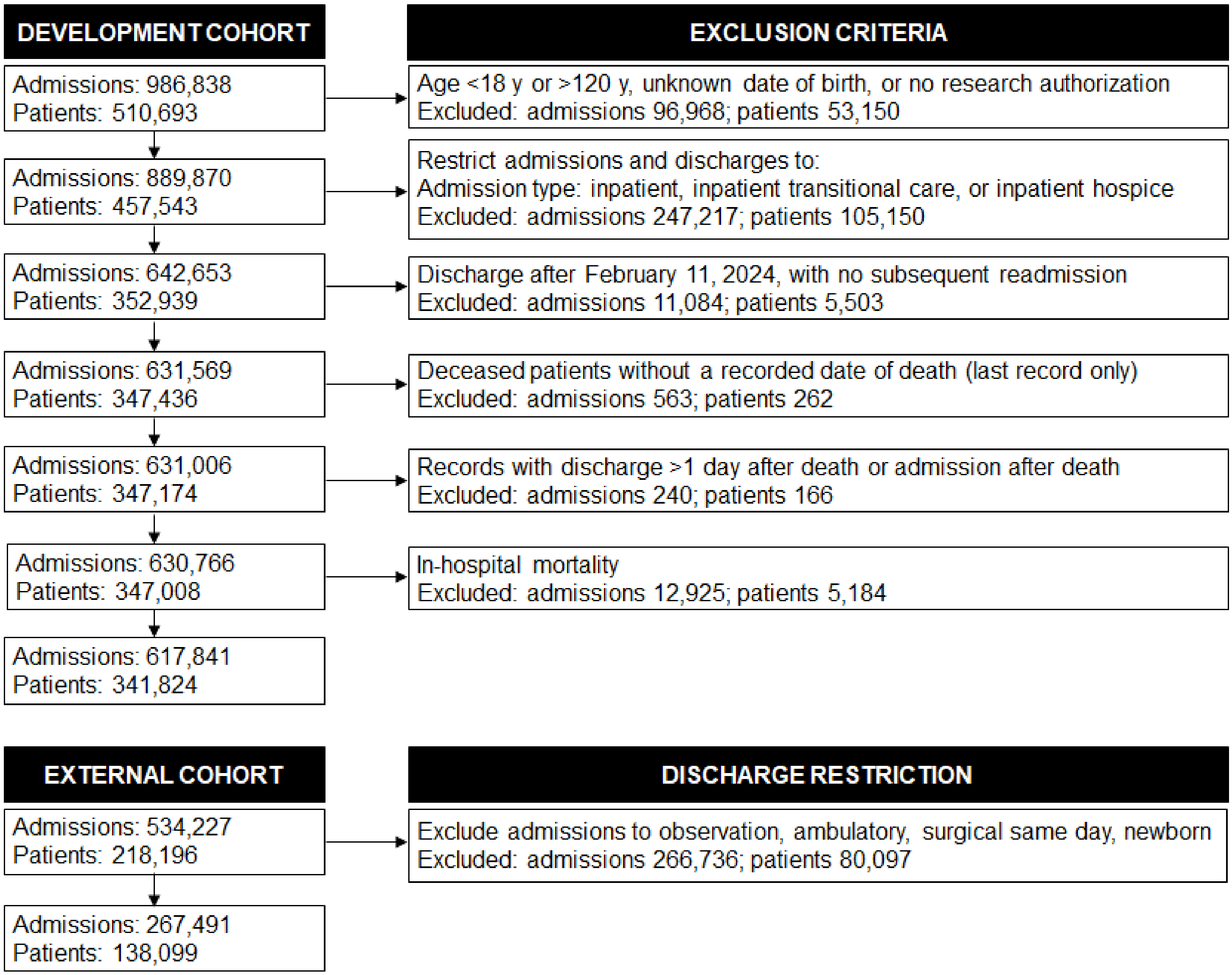

eFigure 1. Cohort Selection for Development and External Cohorts

Flow diagram showing the selection of admissions and patients for the Development and External cohorts. Sequential inclusion and exclusion criteria were applied to identify eligible adult inpatient admissions, including restrictions on age, admission type, discharge timing, and data completeness. The final analytic cohorts included 617,841 admissions (341,824 patients) in the Development cohort and 267,491 admissions (138,099 patients) in the External cohort.

© 2026 Alkayali MZ et al. *JAMA Network Open*.

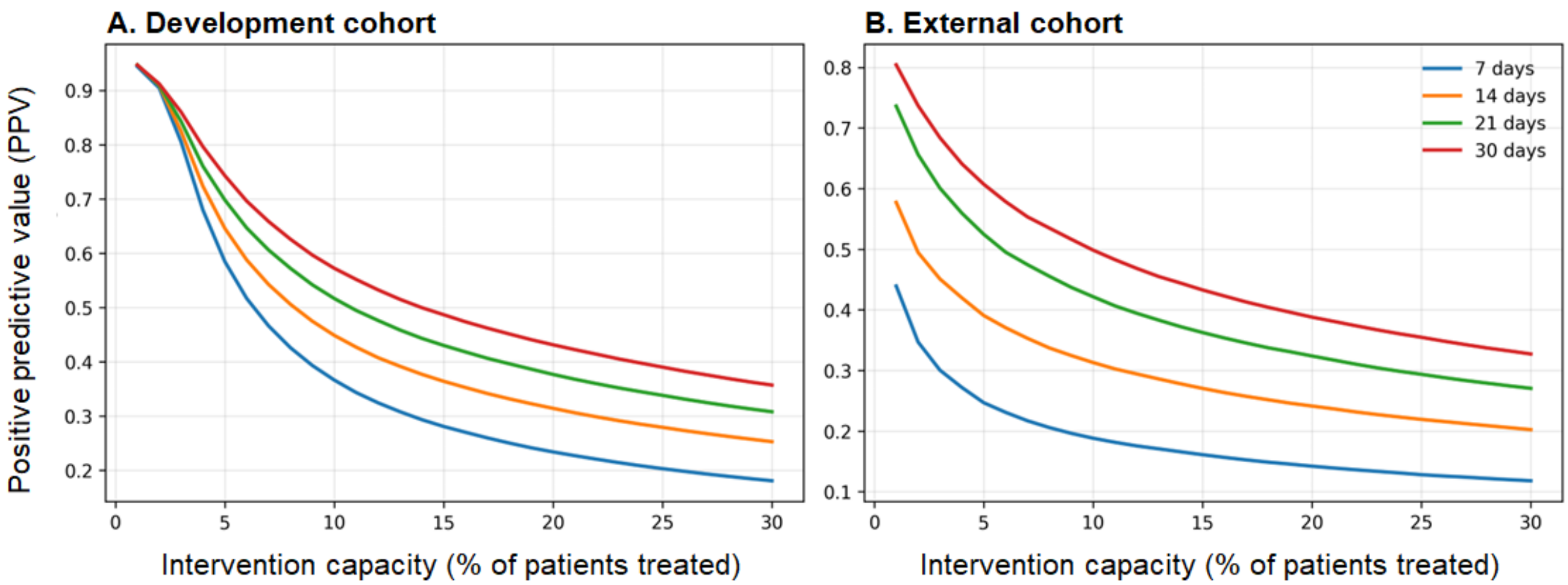

eFigure 2. Positive Predictive Value Across Intervention Capacity and Prediction Horizons

Cumulative positive predictive value (PPV) of readmission risk prediction is shown across increasing intervention capacity at 7, 14, 21, and 30 days after discharge in the development cohort and external cohort. The x-axis represents the proportion of patients selected for intervention, and the y-axis represents PPV, defined as the proportion of selected patients who experienced readmission by the specified time horizon. Cumulative PPV reflects overall risk enrichment by each horizon.

© 2026 Alkayali MZ et al. *JAMA Network Open*.

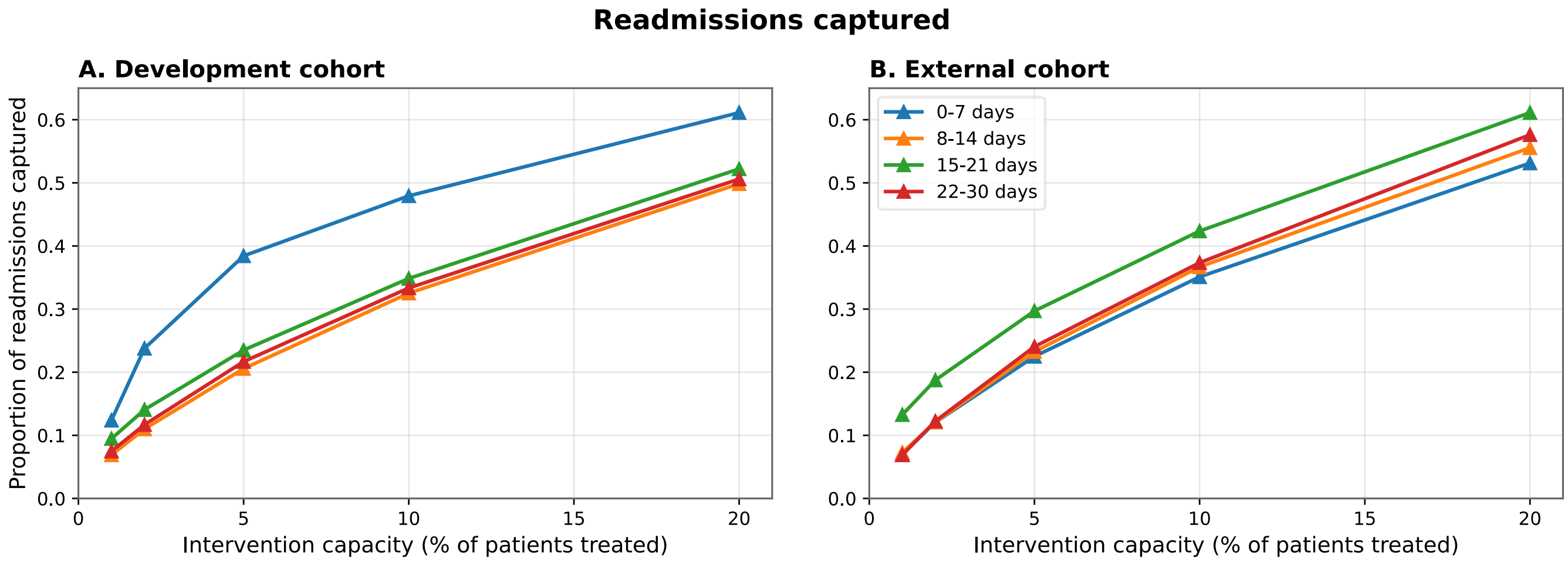

eFigure 3. Landmark Readmissions Captured Across Intervention Capacity

Proportion of interval-specific readmissions captured is shown across intervention capacities for landmark intervals 0-7, 8-14, 15-21, and 22-30 days in the development and external cohorts. Events captured was defined as the proportion of all readmissions during the specified landmark interval that occurred among patients selected for intervention.

© 2026 Alkayali MZ et al. *JAMA Network Open*.

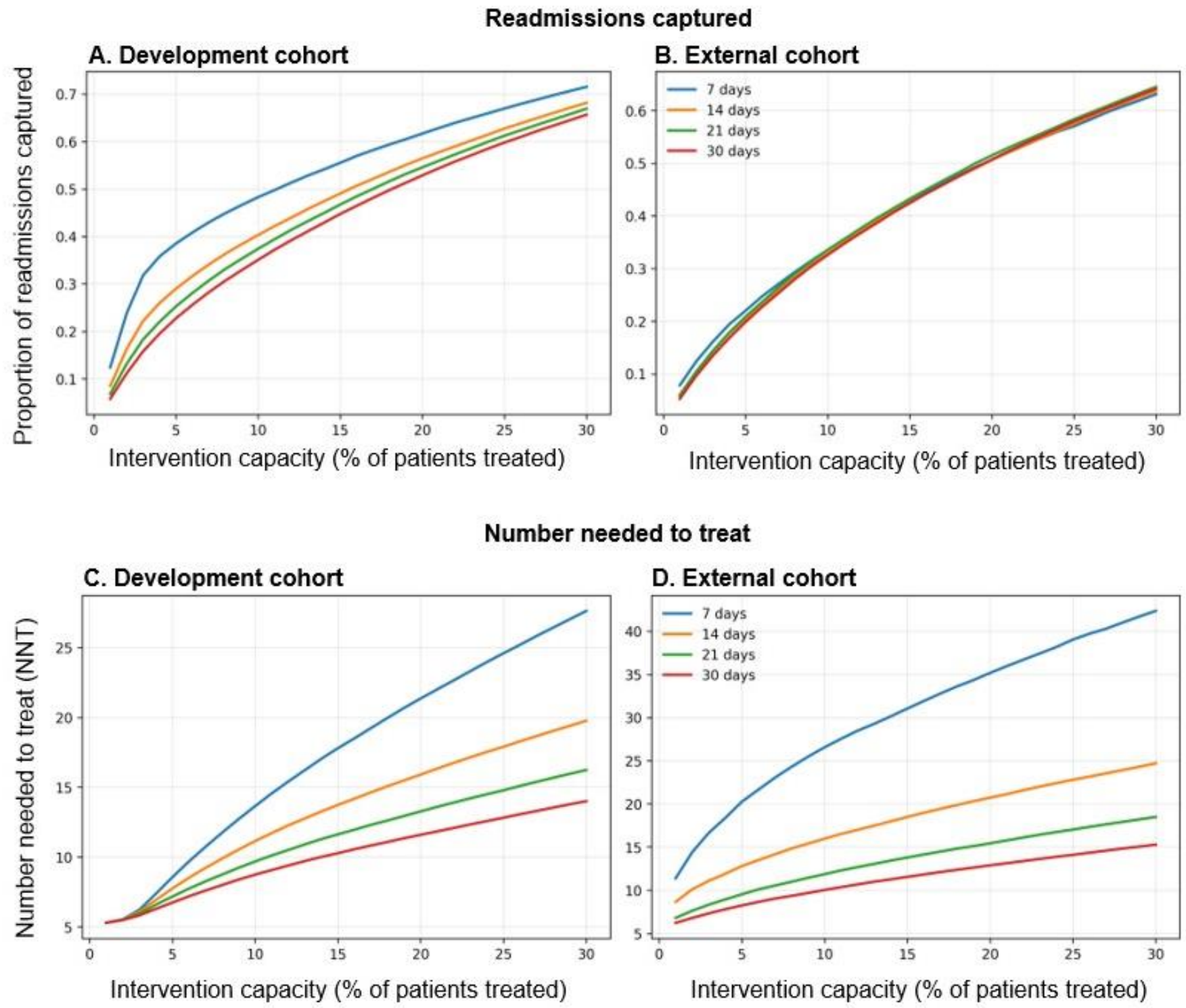

eFigure 4. Cumulative Readmissions Captured and Number Needed to Treat Across Intervention Capacity

(A, B) Proportion of cumulative readmissions captured across increasing intervention capacity in the development and external cohorts. Cumulative readmissions captured was defined as the proportion of readmissions occurring by the specified horizon among patients selected for intervention. (C, D) Cumulative-horizon number needed to treat (NNT) across increasing intervention capacity in the development cohort and external cohort. NNT was calculated from cumulative positive predictive value assuming a 20% relative risk reduction. These cumulative-horizon estimates reflect overall risk enrichment by each horizon.

eReferences

1. Austin PC, Lee DS, Fine JP. Introduction to the Analysis of Survival Data in the Presence of Competing Risks. *Circulation*. Feb 9 2016;133(6):601-9. doi:10.1161/circulationaha.115.017719
2. Chatellier G, Zapletal E, Lemaitre D, Menard J, Degoulet P. The number needed to treat: a clinically useful nomogram in its proper context. *Bmj*. Feb 17 1996;312(7028):426-9. doi:10.1136/bmj.312.7028.426
3. Balasubramanian I, Andres EB, Malhotra C. Outpatient Follow-Up and 30-Day Readmissions: A Systematic Review and Meta-Analysis. *JAMA Netw Open*. Nov 3 2025;8(11):e2541272. doi:10.1001/jamanetworkopen.2025.41272

© 2026 Alkayali MZ et al. *JAMA Network Open*.
