# Supplementary material for: Timing of 30-Day Hospital Readmission Risk and Its Implications for Clinical Utility
Source: JAMA Netw Open. 2026 Aug 13;9(8):e2628846. doi: 10.1001/jamanetworkopen.2026.28846 (PMC13474041; doi:10.1001/jamanetworkopen.2026.28846)
Supplement: Supplementary file 2 [file jamanetwopen-e2628846-s002.pdf]

Data Sharing Statement

Alkayali. Timing of 30-Day Hospital Readmission Risk and Its Implications for Clinical Utility. *JAMA Netw Open*. Published August 13, 2026. doi:10.1001/jamanetworkopen.2026.28846

Data
Data available: No
